# Efficacy of Diterpene Polyalthic Acid Combined with Amphotericin B against *Leishmania amazonensis* In Vitro

**DOI:** 10.3390/ph17091243

**Published:** 2024-09-21

**Authors:** Ana Carolina Bolela Bovo Candido, Mariana Cintra Pagotti, Daiane Albino dos Santos, Lucas Antonio de Lima Paula, Rodrigo Cássio Sola Veneziani, Jairo Kenupp Bastos, Sérgio Ricardo Ambrósio, Lizandra Guidi Magalhães

**Affiliations:** 1 Research Group on Natural Products, Center for Research in Sciences and Technology, University of Franca, Avenida Dr. Armando Salles of Oliveira, Franca 14404-600, SP, Brazil; carolbolela@gmail.com (A.C.B.B.C.); marianacintrap@gmail.com (M.C.P.); lucasalpaula@gmail.com (L.A.d.L.P.); rodrigo.veneziani@unifran.edu.br (R.C.S.V.); sergio.ambrosio@unifran.edu.br (S.R.A.); 2 Animal Science Post Graduation, University of Franca, Franca 14404-600, SP, Brazil; daianealbino.biomed@gmail.com; 3 School of Pharmaceutical Sciences of Ribeirão Preto, University of São Paulo, Av. do Café, s/n, Ribeirão Preto 14040-903, SP, Brazil; jkbastos@fcfrp.usp.br

**Keywords:** amphotericin B, antiparasitic, diterpene, *Leishmania amazonensis*, polyalthic acid

## Abstract

**Background/Objectives:** Leishmaniasis, a neglected disease caused by *Leishmania* spp. including *L. amazonensis*, urgently requires new treatments. Polyalthic acid (PA), a natural diterpene from *Copaifera* spp., has previously demonstrated significant antiparasitic potential. This study evaluated the leishmanicidal effects of polyalthic acid (PA), alone and with amphotericin B (AmpB), on *L. amazonensis* promastigote and amastigote forms. **Results:** PA showed significant activity against promastigotes, with 50% effective concentration (EC_50_) values of 2.01 μM at 24 h and an EC_50_ of 3.22 μM against amastigotes after 48 h. The PA and AmpB combination exhibited a synergistic effect on both forms without inducing cytotoxicity or hemolysis. Morphological changes in promastigotes, including vacuole formation and cell rounding, were more pronounced with the combination. **Conclusions:** These findings suggest that PA and AmpB together could form a promising new treatment strategy against *Leishmania* infections, offering enhanced efficacy without added toxicity.

## 1. Introduction

Leishmaniasis is caused by protozoan parasites of the genus *Leishmania* transmitted through the bite of infected female phlebotomine sand fly vectors [1]. Globally, this neglected disease is endemic in many regions of world, especially in tropical and subtropical areas [2]. It is estimated that 700,000 to 1 million new cases occur annually, representing a significant risk for approximately 1 billion people due to its wide geographic distribution [2].

The pathology manifests in various clinical forms, including cutaneous, mucocutaneous, and visceral leishmaniasis, each presenting unique challenges in diagnosis and treatment [1]. Despite extensive efforts to control the spread of the disease, treatment options are limited, often plagued by issues such as drug resistance, toxicity, and the high cost of existing therapies [2,3,4]. Current first-line treatments for leishmaniasis, pentavalent antimonials, such as sodium stibogluconate and meglumine antimoniate, suffer from drawbacks including the emergence of resistance and adverse side effects [3,4]. The second line of treatment consists of amphotericin B (AmpB) and its formulations, which are also toxic and necessitate intravenous administration [3,4].

**Figure 1:**
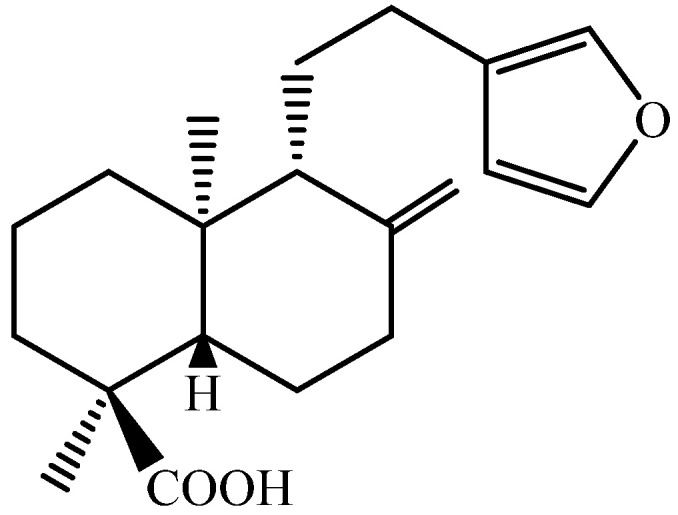
Chemical structure of PA.

Consequently, there is a pressing need to explore alternative treatment strategies that are more effective, safer, and affordable. Approaches aimed at enhancing leishmaniasis therapy encompass the exploration of novel drugs, repurposing of existing medications, and the utilization of combination therapy [5,6]. In this context, polyalthic acid (PA) (Figure 1), a diterpene found abundantly in the oil resin derived from *Copaifera* trees, which are extensively used in Brazilian folk medicine for their diverse therapeutic benefits, has shown notable leishmanicidal activity [7,8,9]. However, previous studies have not investigated the leishmanicidal effect of PA in combination with AmpB, despite combination drug therapy being one of the most rational and promising approaches. This approach offers advantages such as reduced toxicity, synergistic effects, the limited development of drug resistance, a low treatment failure rate, and shorter treatment regimens [5,9]. Therefore, the aim of this study was to evaluate the effects of PA alone and in combination with AmpB, the standard treatment for leishmaniasis.

## 2. Results

### 2.1. PA Exhibits Leishmanicidal Activity against Both Promastigote and Amastigote Forms of L. amazonensis without Causing Cytotoxic or Hemolytic Effects In Vitro

PA and AmpB were evaluated alone to contrast these findings with those achieved using a combination strategy. The 50% effective concentration (EC_50_) values for PA were determined to be 2.01 µM for promastigotes and 3.22 µM for amastigotes at 24 and 48 h, respectively (Table 1). For AmpB, the EC_50_ values were determined as <0.025 µM and 0.095 µM for promastigote and amastigotes, at 24 and 48 h, respectively (Table 1). At 6 and 12 h, EC_50_ values for PA were determined to be higher than 9.35 µM for promastigotes (data not shown).

Cytotoxic and hemolytic assays revealed that PA exhibited lower cytotoxic and hemolytic effects on the peritoneal macrophages and erythrocytes [50% cytotoxic concentration (CC_50_) > 100 µM and 50% hemolytic concentration (CH_50_) > 50 µM] compared to AmpB (CC_50_ 22.41 µM and CH_50_ < 0.195 µM) under the experimental conditions evaluated in this study (Table 1). It was not possible to calculate the selectivity index (SI) for PA due to the CC_50_ value exceeding >100 µM. For AmpB, the SI suggested that it was more effective against amastigotes than cytotoxic (Table 1).

### 2.2. The Combination of PA and AmpB Displays Synergistic Leishmanicidal Activity, While It Results in Indifferent Cytotoxic and Hemolytic Effects In Vitro

PA and AmpB were combined in varying ratios, and the EC_50_ values acquired for each of these drug combinations were employed to calculate the fractional inhibitory concentration (FIC) index and the sum of the FIC (ΣFIC) (Table 2). The combination exhibited a synergistic effect, resulting in an xΣCIF of 0.36 and 0.39 in promastigote and intracellular amastigote forms, respectively (Table 2). Noteworthily, the combination demonstrated an indifferent effect in terms of cytotoxicity and hemolytic effects, resulting in an xΣCIF of 1.39 and 0.68, respectively (Table 2).

### 2.3. The Combination of PA and AmpB Induces Morphological and Ultrastructural Damages in Promastigote Forms of L. amazonensis

**Figure 2:**
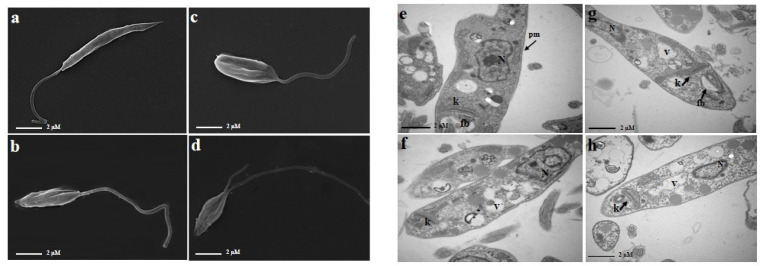
Morphological and ultrastructural alterations in promastigotes of *L. amazonensis* incubated with PA, AmpB, and PA + AmpB for 24 h, analyzed by scanning electron microscopy (SEM) (**a**–**d**) and transmission electron microscopy (TEM) (**e**–**h**). (**a**,**e**) Negative control of promastigotes; (**b**,**f**) samples incubated with PA at EC_50_ (2.01 μM); (**c**,**g**) samples incubated with AmpB at 0.025 μM; and (**d**,**h**) samples incubated with PA + AmpB at EC_50_ (1.62 + 0.021). (fb) flagellar bag; (k) kinetoplast; (pm) plasma membrane; (N) nucleus; (v) vacuole.

The scanning electron microscopy (SEM) analysis observations demonstrated no alteration in promastigote forms of the negative control (incubated with DMSO 0.1%) presenting its typical characteristic of an elongated form (Figure 2a). Conversely, promastigote forms incubated with PA or AmpB alone demonstrated significant alterations in cell volume, the rounding of the cell body shape, and a reduction in flagellum length (Figure 2b,c). Likewise, alterations such as the rounding of the cell body, flagellar transformations, and substantial alterations in the cellular surface were noted in the samples exposed to the drug combination PA and AmpB (Figure 2d).

The ultrastructural observations by transmission electron microscopy (TEM) analysis demonstrated the integrity of the structures of the promastigote forms of the negative control (incubated with DMSO 0.1%) (Figure 2e). Promastigote forms incubated with PA and AmpB alone demonstrated the presence of vacuoles as well as a slight increase in kinetoplasts, damage to the mitochondrion, and small nucleus disorganization (Figure 2f,g). The combination of PA and AmpB showed noticeable alterations such as vacuoles inside the parasites and also swelling in the kinetoplast (arrows) (Figure 2h).

### 2.4. PA and Combination Decrease Cell Volume

**Figure 3:**
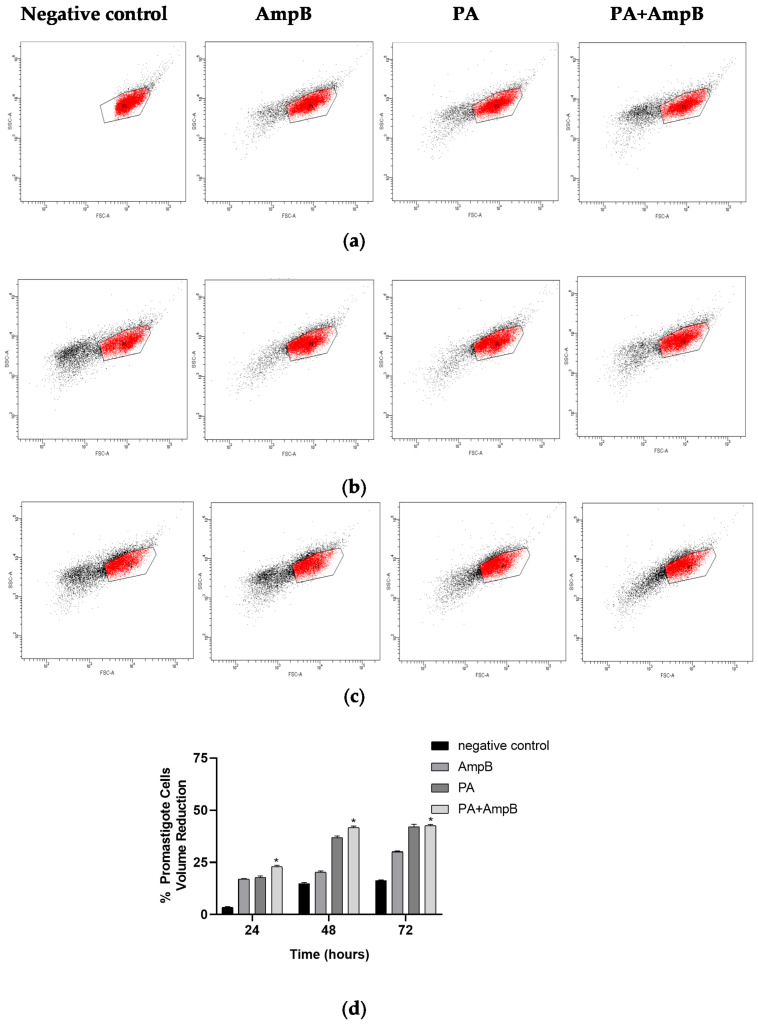
Changes in promastigote volume analyzed by flow cytometer. (**a**–**c**) Corresponding 24 (**a**), 48 (**b**), and 72 h (**c**) of incubation with AmpB at 0.025 μM, with PA at EC_50_ (2.01 μM), and with PA + AmpB at EC_50_ (1.62 + 0.021). Events in red area represent cells with abnormal membrane permeability. (**d**) % of promastigote cells with volume reduction after 24, 48, and 72 hof incubation with AmpB, PA, and combination (PA+ AmpB). * *p* ≤ 0.05, significant difference from negative control.

Promastigote forms incubated with PA and AmpB alone at 24, 48, or 72 h, as well as in combination, demonstrated a time-dependent increase in promastigote forms exhibiting a reduction in cell volume when compared to the negative control group (incubated with 0.1% DMSO) (Figure 3a–d). PA exhibited an increase in promastigote forms with a volume decrease of 17.2%, 36.2%, and 41.1% at 24, 48, and 72 h, respectively. Conversely, AmpB caused an increase in promastigote forms with a volume decrease of 16.8%, 19.8%, and 29.8% at 24, 48, and 72 h. The combination of PA and AmpB also demonstrated a notable effect with a reduction of 22.5%, 41.1%, and 42.3% in promastigote forms with reduced volume in the same time period (Figure 3a–d).

## 3. Discussion

Although there is available treatment for leishmaniasis, the options can be limited particularly for some forms of the disease [2,3,4]. Additionally, concerns related to the toxicity of certain drugs, complex treatment regimens, and the emergence of drug resistance highlight the importance of seeking new therapeutic alternatives for leishmaniasis treatment [3,4]. In this context, compounds derived from natural products are demonstrating considerable potential as alternative treatments for leishmaniasis [10,11,12,13]. Moreover, combination therapies that incorporate both traditional antiparasitic drugs and new compounds with complementary mechanisms of action have the potential to enhance treatment effectiveness while reducing the risk of drug resistance [5,14]. On this basis, the decision to investigate PA was triggered by a previous study that demonstrated the leishmanicidal effect of PA against different species of *Leishmania* [7,8]. AmpB was chosen for combination with PA due to its well-established mechanism of action and efficacy in severe cases of cutaneous leishmaniasis [3,14,15]. This combination represents a strategic approach to maximizing the benefits of AmpB while mitigating its limitations, such as toxicity and resistance, by incorporating the complementary effects of PA.

The effectiveness of PA and AmpB against *L. amazonensis* was initially assessed on promastigote forms, followed by evaluations on amastigote forms and toxicity assessments. In both promastigote and amastigote forms, PA and AmpB demonstrated an EC_50_ lower than 10 µM when evaluated alone, without inducing cytotoxicity or hemolysis under the tested in vitro conditions. According to the criteria for identifying potential hits and leads against leishmaniasis, a hit compound should have an EC_50_ value lower than 10 μM when assessed against the intracellular form of *Leishmania* spp. [16]. Hence, based on the findings for both the amastigote and promastigote forms of *L. amazonensis*, PA appears promising as a potential compound for treating leishmaniasis.

Studies have pointed out the variability in susceptibility to leishmanicidal drugs, such as antimonials, which can vary significantly among species and even among geographically distant strains of the same *Leishmania* spp. [17,18]. In this regard, a previous investigation reported an EC_50_ of 47.46 and 27.43 μM for PA against amastigote forms of *L. amazonensis* (Josefa strain) and *L. donovani*, respectively, which was higher than the value observed in the current study [7,8]. Conversely, AmpB exhibited similar efficacy against *L. amazonensis* (same strain evaluated in this study) [19] but showed variable susceptibility with different strains [20,21].

An essential consideration is the in vitro safety of the drug, as indicated by cytotoxicity and hemolysis assessments. When comparing the drug’s toxicity on peritoneal macrophages with its leishmanicidal effectiveness, it becomes apparent that PA demonstrates lower cytotoxicity compared to its effect on promastigote and amastigote parasite forms. This study further reveals that neither PA nor the combination exhibits relatively indifferent cytotoxic and hemolytic activities, consistent with previous findings by Santos et al. (2013) [7]. In contrast, while AmpB is a cornerstone drug in leishmaniasis treatment, a significant drawback is its requirement for intravenous or intramuscular administration [22,23]. Notably, AmpB is associated with hemolytic activity, observed in this study at concentrations lower than 0.19 µM.

The combination of PA and AmpB demonstrated synergistic effects against both promastigote and amastigote forms while showing an indifferent effect against peritoneal macrophages and erythrocytes. Previous studies have highlighted the potential of combining AmpB with various drugs against *L. amazonenis* [24,25,26]. Consequently, employing combination therapy represents a significant strategy for enhancing the efficacy of existing medications with diverse mechanisms of action in treating various disorders, especially in combating infectious diseases [5,9]. Utilizing different drugs may offer increased effectiveness in treating diseases for several reasons: the combination of drugs enhances efficacy while utilizing lower dosages, potentially reducing toxicity and side effects; impacting numerous cellular pathways or targets delays the emergence of resistant strains, making it challenging to develop resistance against multiple mechanisms [5].

In an effort to understand the impact of combined compounds on the parasite *L. (L.) amazonensis*, experiments were conducted to examine the morphology of the parasites and assess certain oxidative stress markers. SEM analysis revealed that promastigote forms incubated with PA and AmpB showed a reduction in their form, with more pronounced changes observed in the combination incubation. These observations are further supported by findings from the flow cytometry analysis of both isolated compounds and their combinations. Previous research demonstrated that SEM analysis with diterpene hydroxycopalic acid also induced notable morphological alterations, including the emergence of rounded cells, plasma membrane rupture, and significant changes in the flagellar membrane [7].

In TEM analyses, it was observed that promastigote forms, when incubated in combination, exhibited increased kinetoplast size, a higher presence of vacuole formation, mitochondria alteration, and the presence of lipid bodies. However, no alteration or loss of cell membrane integrity was noted under any evaluated condition. Previous research also observed mitochondrial swelling in *L. amazonensis* incubated with hydroxycopalic diterpene [7]. Other studies on epimastigote forms of *Trypanosoma cruzi*, conducted by Izumi et al. (2012) [9], also showed that incubation with diterpene copalic acid induced ultrastructural changes in the parasite such as mitochondrial swelling in almost all cells, while those incubated with hydroxycopalic acid induced organelle disorganization, along with vacuole formation throughout the parasite’s body.

In this context, the mechanism of action for the combination of PA and AmpB remains unclear. AmpB binds to ergosterol, a critical component of the parasite’s cell membrane, creating pores and disrupting membrane function [3,14,15]. Meanwhile, PA appears to affect internal structures such as mitochondria, potentially inducing the generation of reactive oxygen species (ROS) within the parasite. This oxidative stress can damage essential cellular components like proteins, lipids, and DNA [9]. However, further investigations are still necessary to clarify the mechanisms of action involved in the combination.

## 4. Materials and Methods

### 4.1. Compounds

Polyalthic acid (15,16-epoxylabda-8 (17), 13 (16), 14-triene-19-oic acid) (PA) (MW 316.43) (purity estimated between 95 and 98%) was isolated from the oleoresin of *Copaifera duckei* Dwyer, as previously reported by our research group [8,10]. Amphotericin B (AmpB) was purchased commercially from Sigma-Aldrich (St. Louis, MO, USA) (purity ≥97%). For this study, the compounds were previously solubilized in 0.1% dimethyl sulfoxide (DMSO) (Synth).

### 4.2. Parasites and Macrophages

Promastigote forms of *L. amazonensis* (IFLA/BR/67/PH8) were maintained in RPMI 1640 medium (Roswell Park memory Institute)(Gibco^,^- Life Technologies Corporation, Grand Island, NY, USA) supplemented with 10% heat-inactivated fetal bovine serum (FBS) (Gibco), 10 U/mL penicillin, and 10 μg/mL streptomycin (Gibco). The cell culture was maintained in a biological oxygen demand (B.O.D.) incubator at 24 °C. Promastigote forms in the stationary growth phase were used for all of the experiments. Peritoneal macrophages were obtained from male BALB/c mice by washing the peritoneal cavity with cold phosphate-buffered saline (PBS) in RPMI 1640 medium (Gibco) supplement then centrifugated, and cell suspensions were cultivated in RPMI 1640 medium supplement to perform the experiments [27]. 

### 4.3. In Vitro Leishmanicidal Activity

To determine the effect on *L. amazonensis* promastigotes, a suspension containing 1 × 10^6^ promastigotes/mL was placed in a 96-well microplate (Kasvi, São José dos Pinhais, PR, BR) containing RPMI 1640 medium (Gibco) supplement. The parasites were then incubated in the presence of increasing concentrations of PA (0.195 to 50 μM) or AmpB (Sigma-Aldrich) (0.025–1.56 μM) at 25 °C for 6, 12, e 24 h. After each incubation period, parasites were collected aseptically, and viable promastigotes were determined by counting in a Neubauer chamber, taking into account flagellar motility using a light microscope [28,29].

The efficacy of PA and AmpB against *L. amazonensis* intracellular amastigotes was also assessed. To achieve this, a suspension containing 2 × 10^5^ peritoneal macrophages in RPMI 1640 medium (Gibco) supplement was seeded on glass coverslips in a 24-well microplate and incubated for 2 h at 37 °C under 5% CO_2_ to promote cellular adhesion. Following this incubation period, the wells were carefully washed, and adherent macrophages were infected with promastigotes at a concentration of 1 × 10^6^ cells/well (10:1 ratio). This infection process was carried out for 4 h at 37 °C under 5% CO_2_. Subsequently, the wells were washed to remove parasites not internalized in the macrophages, and the infected culture was incubated under the same conditions with different concentrations of PA (1.56 to 25 μM) and AmpB (0.048 to 1.56 μM) for 48. After incubation, the coverslips were fixed with methanol (Synth, Diadema, SP, BR) and stained with 10% Giemsa (Sigma-Aldrich). A total of 200 macrophages were then counted under a light microscope, allowing for differentiation between infected cells and amastigote parasites. The survival index (SI), calculated as the percentage of infected cells multiplied by the average number of amastigotes per infected macrophage, was determined [29].

In both experiments, RPMI 1640 medium (Gibco) with 0.1% of DMSO (Synth) was used as the negative control. The EC_50_ values were determined by nonlinear regression curves. The experiments were performed in triplicate and repeated twice.

### 4.4. Cytotoxicity and Hemolytic Activities

A suspension containing 2 × 10^5^ peritoneal macrophages in RPMI 1640 medium (Gibco) supplement was seeded in a 96-well microplate (Kasvi) and incubated for 24 h at 37 °C under 5% CO_2_ to form a monolayer of confluent cells. After this period, the cells were then incubated in the presence of increasing concentrations of PA or AmpB (Sigma-Aldrich) (0.195 to 100 μM) and incubated again under the same conditions for 24 and 48 h. As a positive control, 25% DMSO (Synth) was employed, and as a negative control, cells were incubated with 0.1% DMSO. After the incubation time, 50 µL of thiazolyl blue tetrazolium bromide (MTT) solution at 2 mg/mL (Sigma-Aldrich) was added to each well and incubated for 4 h. Formazan crystals were solubilized using DMSO, and absorbance was measured using a microplate reader (Libra S12—Biochrom, Cambridge, UK) at 570 nm.

The hemolytic activity was determined as described by Scariot et al. (2017) [30]. An erythrocyte suspension with defibrinated sheep blood (Laborclin, Pinhais, PR, BR) was prepared at a concentration of 6%. The resulting erythrocytes were placed on a 96-well plate in the presence of increasing concentrations of PA or AmpB (Sigma-Aldrich) (0.195 to 50 μM) and incubated for 30 min at 37 °C. Hemolysis was determined by the hemoglobin release of supernatants, and absorbance was measured using a microplate reader at 415 nm (Libra S12—Biochrom). As a positive control, water was employed, and as a negative control, erythrocytes were incubated with 0.1% DMSO. The negative control was erythrocytes with 0.9% NaCl solution, while the positive control used erythrocytes with water.

CC_50_ and HC_50_ were determined by nonlinear regression curves. The experiments were performed in triplicate and repeated twice.

### 4.5. Drug Combination and Determination of Fractional Inhibitory Concentration (FIC) Index

In vitro evaluations of the combinations involving PA and AmpB were conducted using a modified isobologram method [31,32,33]. The concentrations were based on the predetermined 50% concentration [EC_50_ for promastigote (24 h) for PA, amastigote (48 h) for PA and AmpB; CC_50_ for cytotoxicity (48 h) for AmpB] or the concentrations of 0.025 µM for promastigote and 0.195 µM for hemolysis for AmpB and 100 µM and 50 µM for cytotoxicity and hemolysis for PA. Each individual drug was placed in the middle of the serial dilution. It was not possible to calculate CC_50_ and CH_50_ for PA or the EC_50_ of the promastigote and CH_50_ for AmpB; however, the concentrations used at the midpoint of the serial dilution were based as described before. The highest concentrations were established in the proportions of 5:0, 4:1, 3:2, 2:3, 1:4, and 0:5 for PA and AmpB, respectively. These concentrations were then subjected to serial dilution (base 2) until reaching the seventh well on each plate. Subsequently, the parasites, cells, or erythrocytes were added, and the experiments were performed as described previously to determine the EC_50_, CC_50_, or CH_50_ of each combination. The experiments were performed in triplicate and repeated twice.

Following that, the fractional inhibitory concentration (FIC) index of PA and AmpB was calculated by applying the equation EC_50_, CC_50_, or CH_50_ in combination/EC_50_, CC_50_, or CH_50_ of each drug alone or the concentrations described before. The sum of the FIC (ΣFIC) was calculated as follows: ΣFIC = FIC PA+ FIC AmpB [32]. The mean ΣFIC (xΣFIC) was calculated as the average of the ΣFIC. The combinations were considered synergistic for an xΣFIC of 0.5, indifferent with an xΣFIC between 0.5 and 4, and antagonistic for an xΣFIC of 4 [32].

### 4.6. Electron Microscopy

SEM was employed to examine changes in cell surface, while TEM was utilized to investigate alterations in the parasites’ ultrastructure. Promastigote forms (1 × 10^6^/mL) were incubated with PA and/or AmpB at concentrations of EC_50_ alone (2.01 µM to PA and 0.025 µM to AmpB) or EC_50_ in combination for 24 h. After incubation, the parasites were centrifuged and fixed with 3% glutaraldehyde (Sigma-Aldrich) in 0.1 M PBS (pH 7.2) for 24 h. After fixation, the parasites were washed twice with 0.1 M PBS and processed for SEM and TEM as described by [30]. The samples were analyzed in a Joel JSM-5200 scanning electron microscope (Jeol, Tokyo, JP) or in a JEOL Model JEM-100CXII transmission electron microscope (Jeol). As a negative control, a parasite was used in RPMI 1640 (Gibco) medium containing 0.1% DMSO.

### 4.7. Cell Volume Analysis

The cell volume of promastigote forms was evaluated after incubation with PA and/or AmpB for 24, 48, and 72 h at concentrations of EC_50_ alone (2.01 µM to PA and 0.025 µM to AmpB) or in combination according to Santos et al. (2013) [7]. After the incubations, the parasites were then centrifuged at 470 g for 10 min, washed twice, resuspended in 200 μL PBS, and subsequently evaluated by flow cytometry (cell volume analysis) The cells were then counted and analyzed by flow cytometry (BD FACSCanto II) (Franklin Lakes, NJ, USA) and FACSDiva Version 6.1.3 software with 10,000 events for each sample. As a negative control, RPMI 1640 medium (Gibco) containing 0.1% DMSO was used.

### 4.8. Statistical Analyses

The results are presented as the mean of at least three experiments in triplicate ± standard deviation (SD). Non-parametric data underwent analysis using the one-way ANOVA test, and significant differences among means were determined using Dunnett’s test. The EC_50_, CC_50_, and CH_50_ values were determined by nonlinear regression curves. All analyses were performed using GraphPad Prism version 8.0 software (GraphPad software, La Jolla, CA, USA). The SI was calculated by dividing the cytotoxic concentration (CC_50_) by its effective concentration (EC_50_).

## 5. Conclusions

In conclusion, this study demonstrated that PA exhibited potent activity on its own, with promising EC_50_ values, and showed even greater efficacy when combined with AmpB. This combination not only enhanced antiparasitic effects but also avoided cytotoxicity and hemolysis, indicating a safer therapeutic option. The morphological changes observed in the parasites further support the effectiveness of this approach. These findings suggest that the combination of PA and AmpB offers a synergistic and potentially more effective treatment strategy for combating leishmaniasis, warranting further investigation and development.

## Figures and Tables

**Table 1:** Leishmanicidal activity of PA and AmpB against promastigote and amastigote forms of *L. amazonensis*.

	EC_50_		CC_50_	CH_50_	SI
	Promastigotes (24 h)	Amastigotes (48 h)	Peritoneal Macrophages (48 h)	Erythrocytes (30 min)	
PA	2.01 ± 0.24	3.22 ± 0.35	≥100	≥50	ND
AmpB	≤0.025	0.095 ± 0.012	22.41 ± 0.44	≤0.195	235.89

EC_50_: effective concentration of 50% of parasites. CC_50_: 50% cytotoxic concentration in peritoneal macrophage cells after 48 h. CH_50_: 50% hemolytic concentration after 30 min. Selectivity indexes (SIs) were determined by dividing CC_50_ by EC_50_ of amastigote at 48 h. PA: polyalthic acid. AmpB: amphotericin B. ND: not determined.

**Table 2:** Combination profile of PA and AmpB against *L. amazonensis* (promastigotes and intracellular amastigotes) and mammalian cells (peritoneal macrophages) and erythrocytes.

	PA + AmpB	
	x∑FICI	Profile
Promastigotes	0.36	Synergistic
Amastigotes	0.39	Synergistic
Peritoneal macrophages	1.39	Indifferent
Erythrocytes	0.68	Indifferent

The sum of fractional inhibitory concentrations (FICs) (ΣFIC) was calculated as follows: ΣFIC = FIC PA+ FIC AmpB. The mean ΣFIC (xΣFIC) was calculated as the average of the ΣFIC. The combinations were considered synergistic for an xΣFIC of 0.5, indifferent with an xΣFIC between 0.5 and 4, and antagonistic for an xΣFIC of 4.

## Data Availability

The data presented in this study are available on request from the corresponding author.
